# Effect of overexpression of *SNF1* on the transcriptional and metabolic landscape of baker’s yeast under freezing stress

**DOI:** 10.1186/s12934-020-01503-0

**Published:** 2021-01-07

**Authors:** Lu Meng, Xu Yang, Xue Lin, Huan-Yuan Jiang, Xiao-Ping Hu, Si-Xin Liu

**Affiliations:** 1 College of Food Science and Engineering, Hainan University, Haikou, 570228 People’s Republic of China; 2 Engineering Research Center of Utilization of Tropical Polysaccharide Resources, Ministry of Education, Haikou, 570228 People’s Republic of China; 3 Hainan Key Laboratory of Food Nutrition and Functional Food, Haikou, 570228 People’s Republic of China; 4 College of Science, Hainan University, Haikou, 570228 People’s Republic of China

**Keywords:** Transcriptome, Metabolome, *Saccharomyces cerevisiae*, Freezing stress, *SNF1*

## Abstract

**Background:**

Freezing stress is the key factor that affecting the cell activity and fermentation performance of baker’s yeast in frozen dough production. Generally, cells protect themselves from injury and maintain metabolism by regulating gene expression and modulating metabolic patterns in stresses. The Snf1 protein kinase is an important regulator of yeast in response to stresses. In this study, we aim to study the role of the catalytic subunit of Snf1 protein kinase in the cell tolerance and dough leavening ability of baker’s yeast during freezing. Furthermore, the effects of *SNF1* overexpression on the global gene expression and metabolite profile of baker’s yeast before and after freezing were analysed using RNA-sequencing and untargeted UPLC − QTOF-MS/MS, respectively.

**Results:**

The results suggest that overexpression of *SNF1* was effective in enhancing the cell tolerance and fermentation capacity of baker’s yeast in freezing, which may be related to the upregulated proteasome, altered metabolism of carbon sources and protectant molecules, and changed cell membrane components. *SNF1* overexpression altered the level of leucin, proline, serine, isoleucine, arginine, homocitrulline, glycerol, palmitic acid, lysophosphatidylcholine (LysoPC), and lysophosphatidylethanolamine (LysoPE) before freezing, conferring cells resistance in freezing. After freezing, relative high level of proline, lysine, and glycerol maintained by *SNF1* overexpression with increased content of LysoPC and LysoPE.

**Conclusions:**

This study will increase the knowledge of the cellular response of baker’s yeast cells to freezing and provide new opportunities for the breeding of low-temperature resistant strains.

## Background

Frozen dough technology, which separates the processes of dough production and baking, can break through the constraints of the traditional production method of pasta products with regard to the processing time and cost and can ensure the freshness and stability of pasta products [1]. However, freezing and thawing can damage baker’s yeast (*Saccharomyces cerevisiae*) cell activity and weaken its fermentation ability, negatively affecting the quality of final pasta products and limiting the development and application of frozen dough technology [2]. The differential expression of genes brings about metabolite changes and phenotypic diversity of cells. Yeast cells adapt to stress or changing environmental conditions by initiating special procedures for gene expression [3–6] and metabolic regulation [7–9], which promote stress protection, dynamic balance and survival. However, the information about the response of yeast to freezing stress remains limited.

Snf1, a remarkably conserved serine/threonine protein kinase in eukaryotes, participates in many cellular activities, such as the cell cycle, proliferation, endocytosis, metabolism, and stress [10]. The Snf1 protein kinase is a complex that contains an alpha catalytic subunit Snf1, a gamma regulatory subunit Snf4, and one of three alternative beta regulatory subunits Sip1, Sip2, or Gal83 [11, 12]. Previous studies have shown that the Snf1 catalytic subunit plays a positive role in the response to various environmental stresses, such as heat shock [13], alkaline pH [14], Na^+^, Li^+^, hygromycin B [15], oxidative [16], and genotoxic stresses [17]. Our previous work also showed that overexpression of *SNF1* was effective to improve the *S. cerevisiae* cell resistance to ethanol and high glucose stresses [18]. In contrast, Snf1 serves as a negative regulator in the ER stress response [19]. However, the role of the Snf1 catalytic subunit of baker’s yeast in freezing is unclear.

In this study, to investigate the role of Snf1 in the resistance of baker’s yeast cells to freezing stress, the cell survival rate and dough leavening abilities of the transformant ABY+S, which carried the overexpressed *SNF1* gene, and the parental strain were tested. Furthermore, the global transcriptional and metabolic landscapes of the transformant and the parental strain were compared using transcriptome and metabonomics, respectively, before and after freezing.

## Results

### *SNF1* gene expression and growth properties

**Fig. 1:**
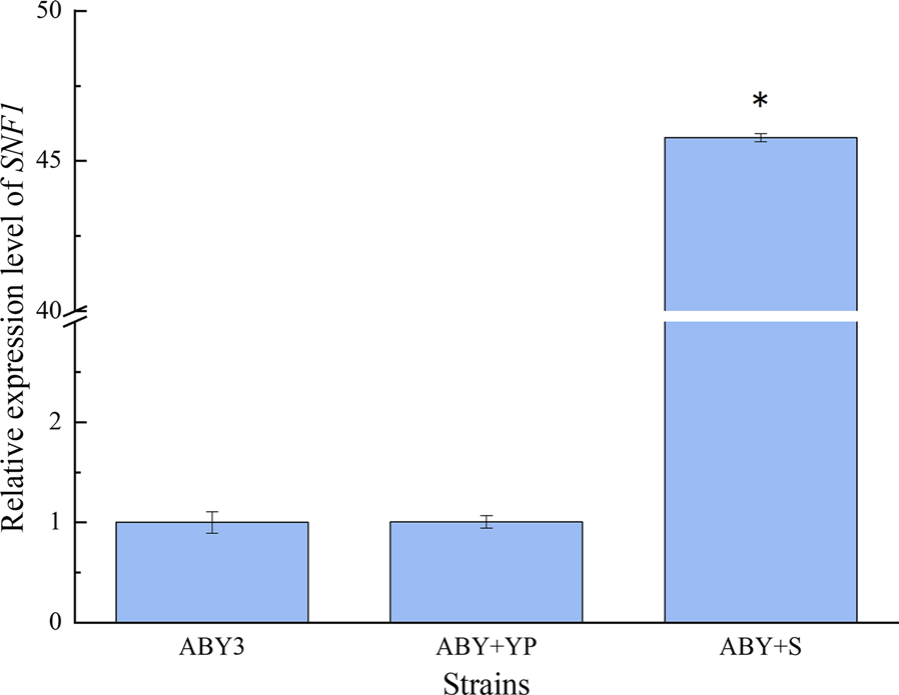
mRNA level of the *SNF1* gene. ABY3: the parental strain; ABY+YP: the strain carrying the vector Yep-PK used as a blank control to demonstrate any possible effect of the empty vector; ABY+S: the transformant carrying *SNF1* overexpression. Significant differences among the three strains (ABY3, ABY+YP, and ABY+S) were confirmed at *p < 0.05

In this study, the transformant ABY+S, which carried the overexpressed *SNF1*, was constructed. Compared to the parental strain ABY3, the mRNA expression level of *SNF1* was upregulated by 45-fold in the transformant ABY+S (Fig. 1). The transformant ABY+YP, which carried the plasmid Yep-PK without target genes was used as a blank control to demonstrate any possible effects of an empty vector and showed similar results to the parental strain.

**Fig. 2:**
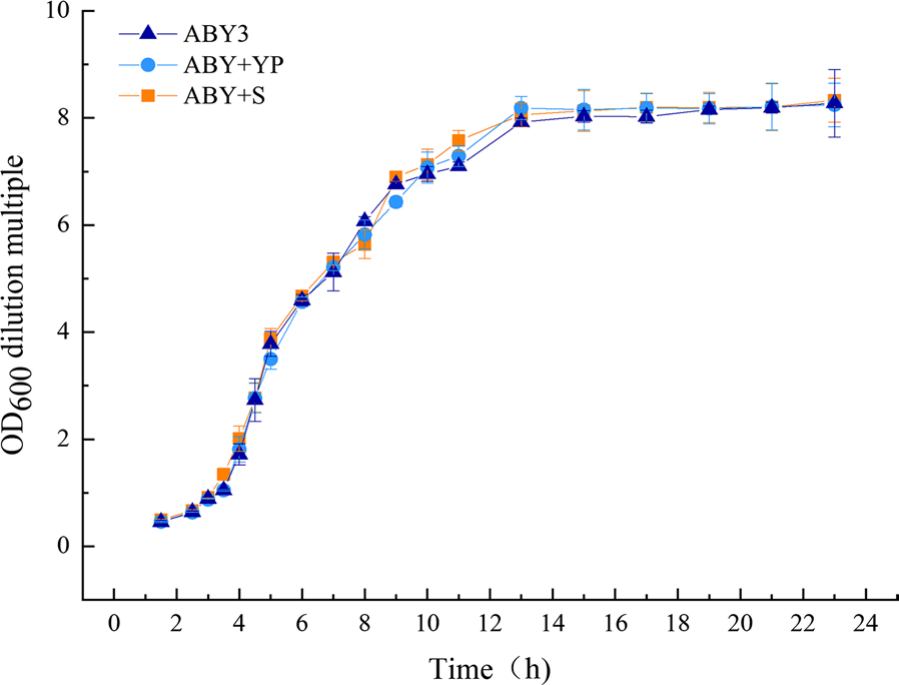
Growth curves of the strains. Growth curves were monitored by measuring the cell density (OD600) at appropriate time intervals. ABY3: the parental strain; ABY+YP: the strain carrying the vector Yep-PK used as a blank control to demonstrate any possible effect of the empty vector; ABY+S: the transformant carrying *SNF1* overexpression

Growth characteristics are essential to the application of industrial strains. To explore the impact of overexpression of *SNF1* in the general growth properties of baker’s yeast cells, the growth curves of the transformants and the parental strain were monitored in YEPD medium. As shown in Fig. 2, no obvious changes were displayed between the transformants and the parental strain. These results suggest that increased *SNF1* gene dosage did not affect the growth properties of the baker’s yeast strain used in this work under general conditions (2% glucose).

### Stress tolerance and leavening ability

**Fig. 3:**
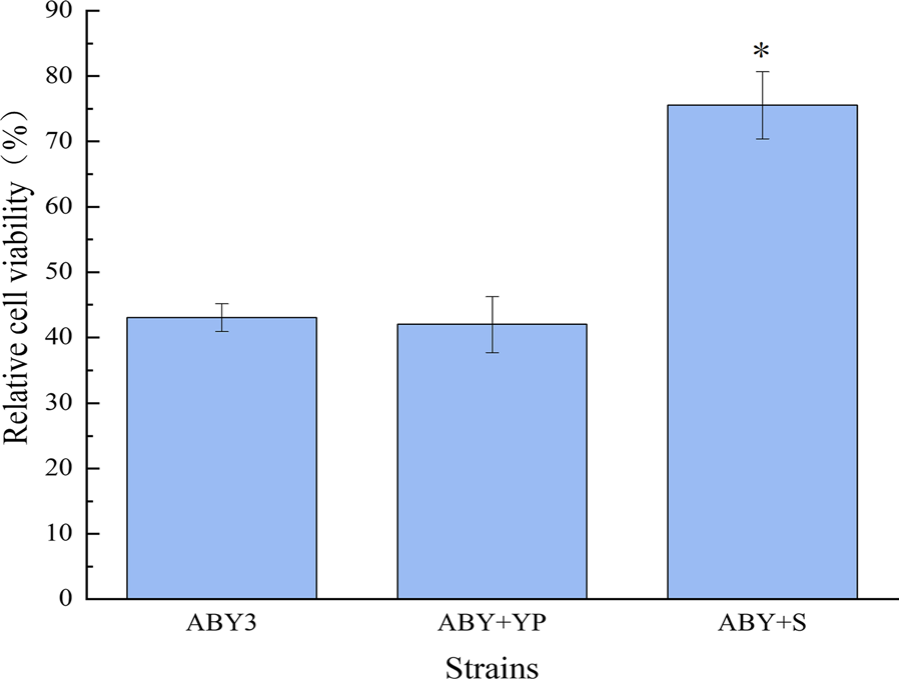
Analysis of freezing tolerance. The cells were cultivated in the LSMLD medium at − 20 °C for 7 days. ABY3: the parental strain; ABY+YP: the strain carrying the vector Yep-PK used as a blank control to demonstrate any possible effect of the empty vector; ABY+S: the transformant carrying *SNF1* overexpression. Significant differences among the three strains (ABY3, ABY+YP, and ABY+S) were confirmed at *p < 0.05

**Fig. 4:**
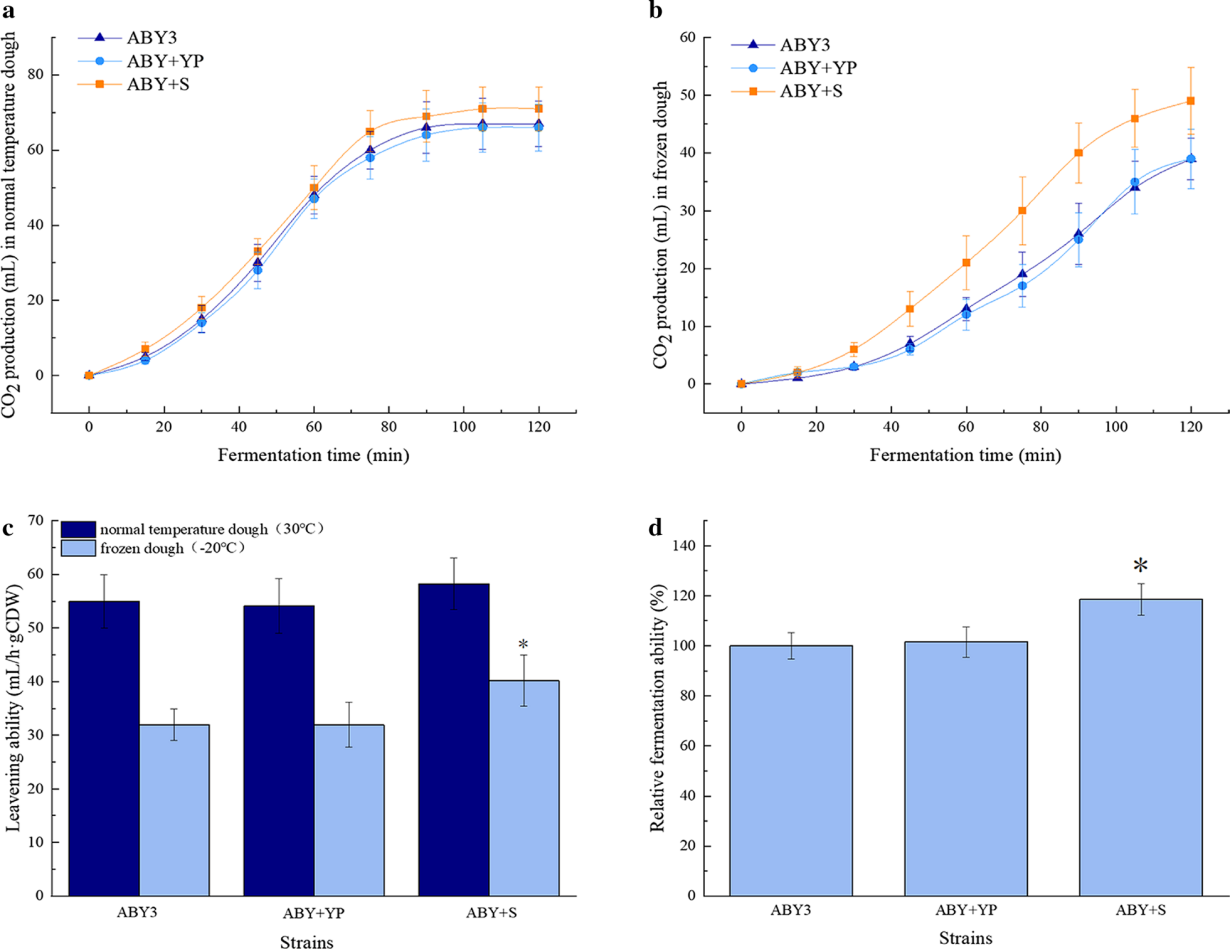
Leavening ability of the strains. **a** CO_2_ production in lean dough before freezing. **b** CO_2_ production in lean dough after freeze-thawing at – 20 °C for 7 days. **c** Leavening ability was determined by the millilitre of increased volume per hour per gram (dry weight) of yeast cells. **d** Relative fermentation ability was determined by the ratio of leavening ability after freeze-thawing to that before stress, and the results were expressed relative to that of the parental strain ABY3 (defined as 100%). ABY3: the parental strain; ABY+YP: the strain carrying the vector Yep-PK used as a blank control to demonstrate any possible effect of the empty vector; ABY+S: the transformant carrying *SNF1* overexpression. Significant differences among the three strains (ABY3, ABY+YP, and ABY+S) were confirmed at *p < 0.05

To evaluate the effect of *SNF1* overexpression of baker’s yeast cells in freezing stress, the cell survival rate and leavening ability were measured. As shown in Fig. 3, compared to the parental strain ABY3, the cell survival rate of the transformant ABY+S was 32% higher than that of the parental strain after 7 days of freezing. Similar behaviour was observed in the control transformant ABY+YP to the parental strain. For the leavening ability, slight changes were observed in the transformant ABY+S and the parental strain ABY3 before freezing (Fig. 4a, c). Nevertheless, the CO_2_ production in 120 min and leavening ability of the transformant ABY+S were 26% and 28% higher than that of the parental strain, respectively, after freezing (Fig. 4b, c), and the relative leavening ability increased by 11% (Fig. 4d). The control transformant ABY+YP displayed no significant differences compared to the parental strain ABY3. Due to *SNF1* deficiency, yeast cell growth is limited under nutrient limitation [10]. In this study, overexpression of *SNF1* increased the cell resistance and improved the fermentation properties of baker’s yeast in freezing, but did not affect the growth in 2%. These findings confirm the importance of Snf1 in stress and unstress conditions.

### Global gene expression

**Fig. 5:**
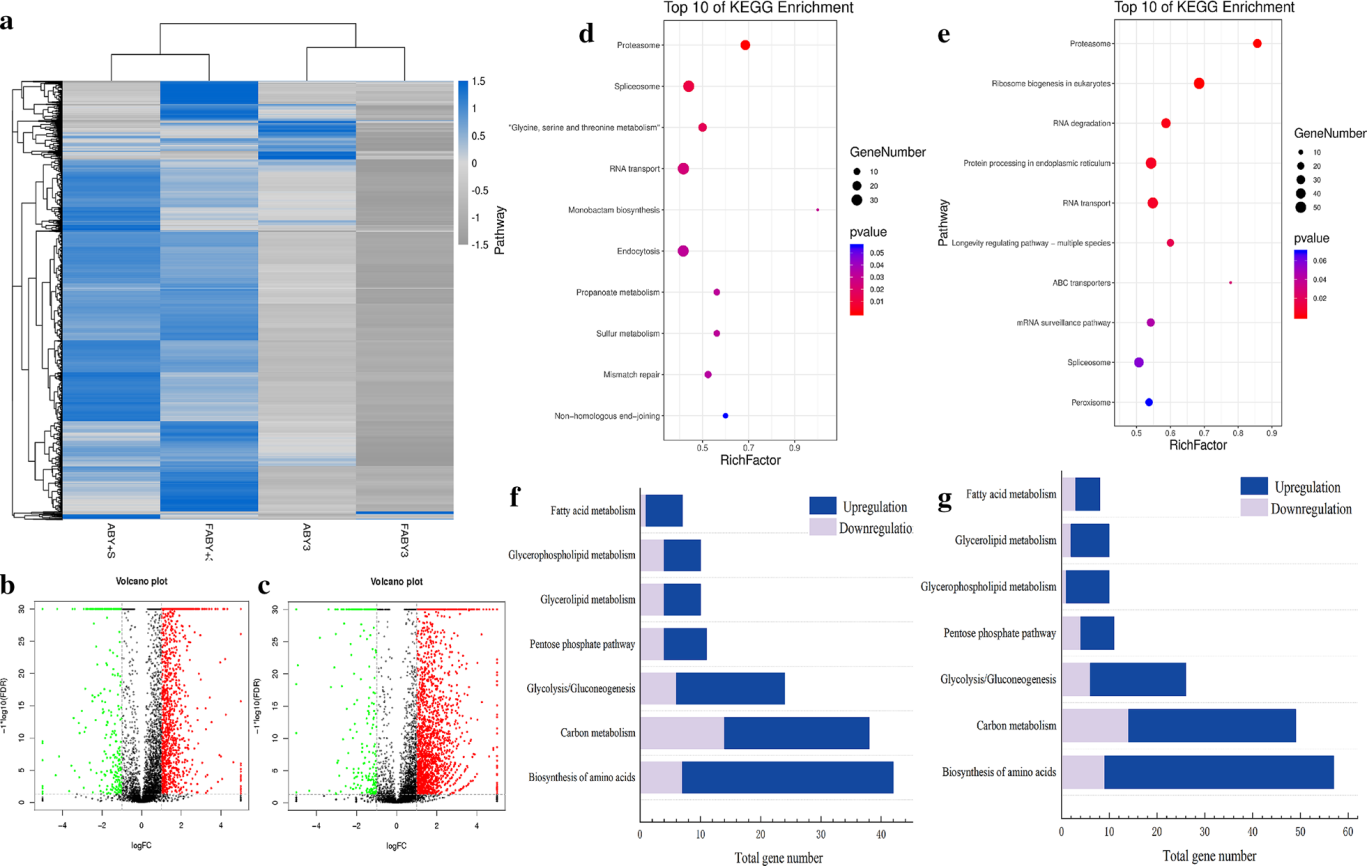
mRNA expression levels and enrichment analysis by transcriptomics. **a** Hierarchical clustering analysis of differentially expressed genes. ABY3 and ABY+S represent the parental strain and the transformant, respectively; F represents freeze-thawing stress. **b** The distribution diagram of up- and downregulated DEGs in the KEGG pathway between ABY3 and ABY+S before freezing stress. **c** Distribution diagram of up- and downregulated DEGs in the KEGG pathway between ABY3 and ABY+S after freezing stress. Red and green indicate upregulation and downregulation, respectively. **d** KEGG enriched pathways of DEGs before freezing stress. **e** KEGG enriched pathways of DEGs after freezing stress. **f** Expression profile of the genes belonging to important metabolic pathways in KEGG before freezing stress. **g** Expression profile of the genes belonging to important metabolic pathways in KEGG after freezing stress. The grey colour shows the genes with significantly reduced transcription levels. The blue colour shows the genes with significantly elevated transcription levels

To explore the effect of *SNF1* overexpression on the global gene expression of baker’s yeast during freezing, RNA-seq and bioinformatic analysis were conducted. The differentially expressed genes (DEGs) of the strains before and after freezing were analysed by cluster analysis of differential expression patterns among the four strains (the parental strains and the transformants ABY+S non-suffered and suffered freezing). As shown in Fig. 5a, compared to the parental strain ABY3, the DEGs of the transformant ABY+S were generally upregulated before and after freezing. The k-means clustering of all DEGs indicated that the strains were obviously divided into two groups, and the gene expression pattern of the transformant ABY+S was significantly different from that of the parental strain ABY3. According to Fig. 5b, 1799 genes showed significant differences between the transformant ABY+S and the parental strain ABY3 before freezing, of which 1453 genes were upregulated and 346 downregulated. With the same analysis method, as shown in Fig. 5c, 2308 genes were significantly different between the strains after freezing (namely, FABY+S and FABY3), of which 2035 genes were upregulated and 273 genes downregulated.

The KEGG enrichment analysis of the significantly differentially expressed genes of the strains before and after freezing showed that overexpression of *SNF1* had the greatest effect on the proteasome pathway, especially after freezing (Fig. 5d, e). The function of the proteasome involves the cell cycle, DNA repair, apoptosis, gene transcription, stress response, and signal transduction [20]. When cells respond to stress, proteasomes are responsible for the degradation of intracellular misfolded proteins labelled by heat shock proteins, which directly affects the renewal of misfolded proteins and ensures related biological function [21]. In addition, the DEGs enrichment pathways before freezing included spliceosome and RNA transport; ribosome biogenesis in eukaryotes, RNA degradation, endoplasmic reticulum protein processing, RNA transport, mRNA surveillance pathway, and spliceosome were included in the DEGs enrichment pathways after freezing. Many gene transcriptional regulation and protein processing and regulatory pathways were affected by *SNF1* overexpression. The peroxidase pathway of the transformant ABY+S significantly differed from that of the parental strain ABY3 after freezing. When yeast is subjected to freezing–thawing stress, the metabolism of free radicals in cells is destroyed. The accumulation of free radicals can cause or aggravate lipid peroxidation in cells, resulting in damage to the membrane system and macromolecules in the body. The regulation of the peroxidase pathway, which is related to the regulation of cell stress response, can alleviate this damage [22, 23].

The frozen survival rate and fermentation performance embody the ability of baker’s yeast cells to self-protect and remain vivacious. The metabolic pathways related to cell self-protection and nutrient consumption were screened from the KEGG enrichment pathway before and after freezing, including carbon metabolism, biosynthesis of amino acids, glycolysis/gluconeogenesis, pentose phosphate pathway, glycerolipid metabolism, glycerophospholipid metabolism, and fatty acid metabolism. The differences in the expression of DEGs related to the above metabolic pathways before and after freezing were analysed (Fig. 5f, g). Carbon metabolism is the most important process of biological growth and metabolism and is strictly regulated by environmental signals. Varying degrees of cell pathological changes and death can occur as a result of carbon metabolism disorders [24]. Like most eukaryotes, the carbon metabolism of baker’s yeast mainly includes glycolysis/gluconeogenesis, TCA cycle, the pentose phosphate pathway, and the glyoxylate cycle, which directly affect the fermentation ability of strains. According to the statistics of the pathway database, 115 genes were annotated in the carbon metabolism pathway. In the pre-fermentation phase before freezing, 38 DEGs in this pathway were significantly different between the transformant ABY+S and the parental strain ABY3, of which 24 DEGs were upregulated and 14 DEGs downregulated. In the carbon metabolism pathway, 24 DEGs were involved in the glycolysis pathway, of which 18 were upregulated and 6 downregulated. 11 DEGs were involved in the pentose phosphate pathway, of which 7 DEGs were upregulated and 4 downregulated. After freezing, 49 DEGs showed significant differences in carbon metabolism between the transformant ABY+S and the parental strain ABY3, of which 35 DEGs were upregulated and 14 downregulated. Among them, 26 DEGs were involved in the glycolysis pathway, of which 20 DEGs were upregulated and 6 downregulated. 11 DEGs were involved in the pentose phosphate pathway, of which 7 DEGs were upregulated and 4 downregulated.

As the raw material for the synthesis of proteins and enzymes, intracellular amino acids are important substances for cell growth and metabolism. Some studies have shown that yeast has a preference for organic nitrogen sourced amino acids in low temperature fermentation, and the content of intracellular amino acids in yeast is generally downregulated after freezing stress [25–27]. Expression of the amino acid biosynthesis pathway is helpful to supplement intracellular amino acid deficiency in a low-temperature environment. According to the statistics of the pathway database, 125 genes were annotated in the amino acid biosynthesis pathway. In the pre-fermentation phase before freezing, 42 genes of the transformant ABY+S were significantly different from the parental strain ABY3, of which 35 DEGs were upregulated and 7 DEGs downregulated, while 57 genes were significantly different in fermentation after freezing stress, of which 48 DEGs were upregulated and 9 DEGs downregulated.

The HOG-MAP kinase pathway is involved in the glycerolipid metabolism. Under freeze–thaw stress, *S. cerevisiae* activates the HOG-MAP pathway by reducing membrane fluidity. Glycerol in this pathway participates in osmotic regulation and adaptation to the low temperature growth of yeast and balances the ratio of intracellular NADH/NAD, which can be used as antifreeze [28]. According to the statistics of the pathway database, 27 genes were annotated in glycerolipid metabolism. In the pre-fermentation phase before freezing, the transformant ABY+S showed significant differences compared to the parental strain ABY3, of which 6 DEGs were upregulated and 4 DEGs downregulated. In the fermentation phase after freezing stress, 10 genes showed significant differences, of which 8 DEGs were upregulated and 2 DEGs downregulated.

Glycerophospholipid and fatty acid metabolism are related to cell membrane composition. The plasma membrane performs a series of physiological functions in the cell, including morphological regulation and information transmission. The function of the membrane is related to the proportion of the membrane components [29]. According to the statistics of the pathway database, 38 genes were annotated in the glycerophospholipid metabolism. The transformant ABY+S was compared with the parental strain ABY3 in the pre-fermentation phase before freezing, of which 6 DEGs were upregulated and 4 DEGs downregulated. In the fermentation phase after freezing stress, 10 genes showed significant differences, of which 9 DEGs were upregulated and 1 DEG downregulated. Overall, 21 genes were annotated in fatty acid metabolism when comparing the transformant ABY+S with the parental strain ABY3 in the pre-fermentation phase before freezing, including 6 upregulated DEGs and 1 downregulated DEG. 8 genes showed significant differences in fermentation after freezing stress, of which 5 DEGs were upregulated and 3 DEGs downregulated.

### Metabolomic profiling

**Fig. 6:**
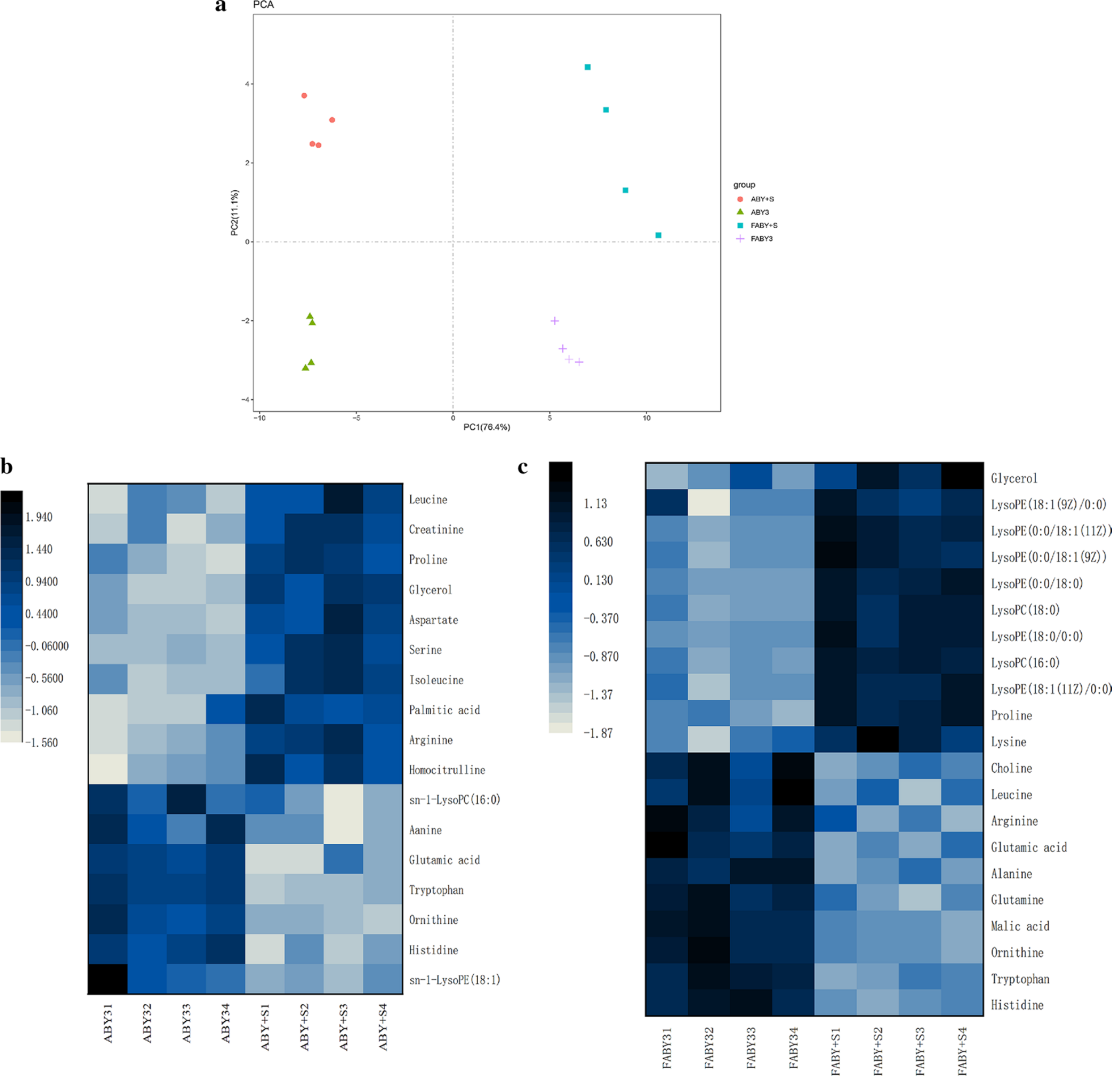
Analysis of differentially expressed metabolites. **a** PCA result of metabolome data. **b** The unsupervised clustering of 17 differential metabolites between the transformant ABY+S and parental strain ABY3 before freezing stress. **c** The unsupervised clustering of 21 differential metabolites between the transformant ABY+S and parental strain ABY3 after freezing stress

In total, 77 metabolites were detected and identified by metabolic analysis in four strains (the parental strains and the transformants ABY+S non-suffered and suffered freezing). PCA result showed that significant differences and repeatability were existed between samples (Fig. 6a). Among them, there were 17 significant differential metabolites in the pre-fermentation before freezing, of which 10 metabolites were upregulated and 7 metabolites downregulated. There were 18 significantly differential metabolites in fermentation after freezing stress, of which 3 metabolites were upregulated and 15 metabolites downregulated.

Compared to the parental strain ABY3, 17 significantly different metabolites were observed in the transformant ABY+S, of which 10 metabolites were upregulated and 7 metabolites downregulated in the pre-fermentation phase before freezing (Fig. 6b). 6 kinds of amino acids included in the 10 upregulated metabolites, among which arginine and proline have clear cryoprotective effects [24, 30]. Related studies have shown that the fermentation ability of proline-accumulating baker’s yeast in frozen dough is higher than that of the wild strain [31]. The content of glycerol, a well-known metabolite as a cell cryoprotectant [32, 33], was significantly upregulated in the transformant ABY+S. In addition, three substances in the differential metabolites were related to cell membrane components: upregulated palmitic acid and downregulated sn-1LysoPC (16:0) and sn-1LysoPE (18:1). As the protective barrier of cells, the cell membrane can maintain cell morphology through lipid changes during environmental adaptation and regulate signal transduction and stress response [28, 34–36]. In the fermentation phase after freezing stress (Fig. 6c), compared to the parental strain ABY3, there were 21 significantly different metabolites in the transformant ABY+S, of which 11 metabolites were upregulated and 10 metabolites downregulated. Protectants lysine, proline, and glycerol are included in the upregulated differential metabolites. Among the downregulated metabolites, LysoPC and LysoPE accounted for a large proportion of the differential metabolites. LysoPC is a kind of bioactive phospholipid that is produced by phosphatidylcholine (PC) hydrolysis mediated by phospholipase in cells [29]. The effect of LysoPC on freezing tolerance of yeast has not been determined.

### Analysis of the enrichment pathway of significantly different genes and metabolites

According to the transcriptome and metabonomic analyses, DEGs and differential metabolites were mainly enriched in the amino acids, glycerolipid, and glycerophospholipid metabolism (Tables 1, 2). During fermentation before freezing stress, compared to the parental strain ABY3, the transformant ABY+S accumulated 6 types of amino acids including leucine, proline, serine, isoleucine, arginine, and homocitrulline, and the expression of genes related to the amino acid synthesis upregulated. The transformant ABY+S accumulated more glycerol than the parental strain. Accordingly, the expression of gene *GPP1* encoding glycerol-3-phosphatase upregulated, which regulates the synthesis of glycerol. During fermentation after freezing stress, only proline and lysine of the transformant ABY+S exhibited more content than the parental strain. The intracellular glycerol of the transformant ABY+S remained high with upregulated expression of *GPP1* and *GPP2* (encoding glycerol-1-phosphatase) but downregulated expression of *GUT2* (encoding glycerol-3-phosphate dehydrogenase) after freezing. The expression of genes involved in glycerol phospholipid metabolism before and after freezing was significantly different between the transformant ABY+S and the parental strain. Simultaneously, the transformant ABY+S and the parental strain showed difference in the accumulation of LysoPC and LysoPE.

**Table 1:** DEGs in the enrichment pathway before freezing

Pathways	Upregulation genes	Downregulation genes
Biosynthesis of amino acids	*CDC19, TKL2, HIS7, LEU2, HIS4, CHA1, PGK1, THR4, GPM2、LYS20, HOM2, SAM2, HOM3, SER3, TRP2, MET6, LEU1, STR3、CYS4, TDH3, PFK1, ARG4, YHR112C, SER33, LYS12, HIS5、TDH1, GPM1, SHM2, MET17, ILV5, PFK2, SER1, GLN1*	*ARO7, HIS1, IRC7, YHR033 W, ARG3, DP2, RKI1*
Glycerolipid metabolism	*YPR1, GPP1, DAK1, GCY1, ALE1, DGA1*	*SCT1, DAK2, GPT2, ALD4*
Glycerophospholipid metabolism	*CHO2, PSD2, SPO14, ALE1, GPD2, CKI1*	*GPT2, GUT2, SCT1, PIS1*

**Table 2:** DEGs in the enrichment pathway after freezing

Pathways	Upregulation genes	Downregulation genes
Biosynthesis of amino acids	*CYS3, CDC19, TKL2, HIS7, HIS4, CHA1, PGK1, THR4, IDP1, PRO1, SAM2, HOM3, SER3, TRP2, MET6, PRS2, ARO2, STR3, NQM1, CYS4, TDH3, SER2, PFK1, ENO1, ARG4, BAT1, SER33, LYS12l HIS5, TDH1, ACO2, TDH2, STR2, HOM6, TRP3, SHM2, MET17, ILV5, ILV2, ERR3, LEU4, PHA2, PRS5, ARG8, ERR1, CAR1, ERR2, GLN1*	*CIT2, HIS1, ARG5,6, YHR033 W, HIS6, ARG3, IDP2, IDP3, CIT3*
Glycerolipid metabolism	*ATG15, TGL2, YPR1, GPP2, GPP1, DAK1, GCY1, DGA1*	*ALD4, DAK2*
Glycerophospholipid metabolism	*CDS1, CHO2, GDE1, OPI3, SPO14, CKI1, PLB2, CPT1, GPD2*	*GUT2*

## Discussion

**Fig. 7:**
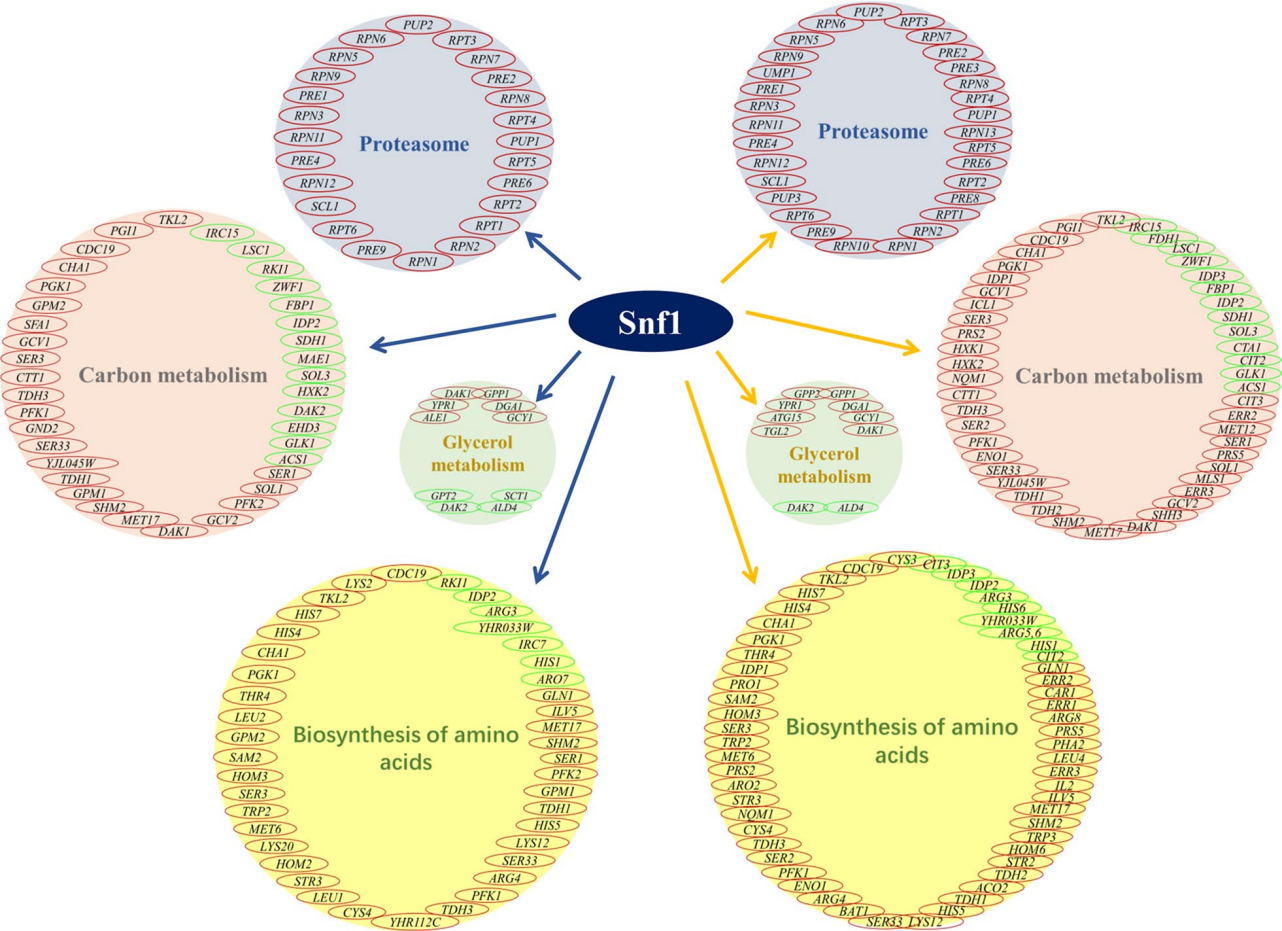
Regulation of Snf1 before and after freezing. The blue arrow points before freezing; The yellow arrow points after freezing; The red and green circles indicate upregulated genes and downregulated genes, respectively

Snf1, a global regulator in yeast, participates in the response to stress in multiple regulatory modes. Casamayor et al. [14] showed that *SNF1* mutation of *Saccharomyces cerevisiae* exhibited an alkali-sensitive phenotype and the role of Snf1 in the resistance to alkaline pH was largely rely on its function in the adaptation to glucose scarcity, with altered trehalose metabolism, glycogen/glucan metabolism, sugar transport, and phosphorylation. In this work, overexpression of *SNF1* increased the cell resistance and leavening ability of baker’s yeast cells during freezing, which could be attributed by the altered transcriptional and metabolic patterns, but not the complete same way in the tolerance to alkalinization (Fig. 7).

Overexpression of *SNF1* had an important regulatory impact in the proteasome. This finding was consistent with the results of Yao et al. [37], who showed that proteasomes and Snf1 interconnected in the regulation of aging. The regulatory impact was more apparent after freezing stress and was characterized by the high expression of a large number of proteasome-related genes (Additional file 1a and b). Yeast cell-controlled proteolysis via the ubiquitin–proteasome system relieves cellular stress through clearing misfolded proteins [20, 21]. Snf1 regulated the expression of proteasome-related genes and enhanced the degradation efficiency of misfolded proteins under freezing stress, achieving the supervision of cell quality. This ensures that the cells still maintain high growth and metabolic activity after freezing injury.

Overexpression of *SNF1* increased carbon metabolism before and after freezing with the high expression of many genes involved in carbon metabolism (Additional file 2a and b). These findings were consistent with the results of Casamayor et al. [14], who found that Snf1 mutation downregulated the expression level of many genes involved in sugar metabolism of yeast induced by alkaline stress. The surviving cells maintained strong growth and metabolism abilities of baker’ yeast, which ensured strong fermentation ability after freezing. Overexpression of *SNF1* could effectively enhance the maltose utilization and dough leavening ability of industrial baker’s yeast [38], which is also reflected after freezing stress. Snf1 regulated glycerol metabolism via the regulation of the transcription factor Adr1 in glucose [39]. In this study, *SNF1* overexpression increased the accumulation of the reserve carbon source and cell protectant glycerol (Additional file 3a and b). This could slow down the damage of yeast cells to a certain extent and improve the survival rate. These findings were consistent with the results of Casamayor et al. [14], suggesting that Snf1 regulated glycerol metabolism in nutrition and environmental stresses.

Before freezing, the accumulation of 6 types of amino acids in *SNF1* overexpression was higher than that of the wild type. The expression of genes related to the biosynthesis of amino acids before freezing was consistent with the metabonomic data (Additional file 4a). Enhanced accumulation of amino acids can improve the stress resistance of cells in coping with freezing. In addition, in the low temperature, extracellular amino acids could be consumed as organic nitrogen sources, which is likely to lead to the lack of available amino acids. The accumulation of intracellular amino acid can provide sufficient raw materials for the synthesis of proteins and enzymes in cells. Compared to the parental strain, overexpression of *SNF1* decreased the accumulation of several amino acid (not including proline and lysine) after freezing. However, the transcriptional results regarding amino acid synthesis was not corresponded with the metabonomic data after freezing (Additional file 4b). Based on the metabolic pathway data, the decreased intracellular amino acids in *SNF1* overexpression after freezing could be caused by the following reasons. First, enhanced carbon metabolism and glycerol synthesis could reduce the amount of carbon frame for the biosynthesis of amino acids by *SNF1* overexpression, weakening the synthesis of amino acids. Second, increased proteasome activity in *SNF1* overexpression could improve the efficiency of misfolded protein degradation and promote the regeneration of proteins, whereas intracellular amino acids, as the raw material of protein synthesis, were consumed more in protein synthesis, resulting in a decrease in intracellular amino acids; but the correction of misfolded proteins is helpful to ensure the normal growth and metabolism of cells, maintaining a strong fermentation performance in frozen dough. The stable accumulation of proline and lysine could contribute to the high survival rate and strong leavening ability in *SNF1* overexpression [40, 41]. The change of each amino acid was not the same in *SNF1* overexpression under different conditions, such as that in freezing and non-freezing in this work and that in high glucose, ethanol, and heat shock stresses of our previous study [33]. In addition, Nicastro et al. [42] showed that Snf1 inhibited the biosynthesis of amino acids in amino acid-rich condition. These results exhibit no contradictory and suggest that the role of Snf1 converted in different signals. The regulation of Snf1 on the metabolism of amino acids might be in a signal-dependent manner. For example, overexpression of *SNF1* promoted the expression of *MSN2*, a transcription factor for the transport of amino acid after freezing, whereas no obvious change was observed before freezing in this work. Snf1 inhibits the translation of Gcn4, a transcription factor of amino acid synthesis in nutritional stress [43], but the regulation of Snf1 on Gcn4 in other stress is not clear. The mechanism of Snf1 regulating amino acids metabolism under different signals needs further study.

Previous studies showed that *SNF1* mutation affected the phospholipid biosynthesis via the alternation of the production and proportion of fatty acids [44, 45]. In this study, overexpression of *SNF1* changed the content of LysoPC and LysoPE in varied degree before and after freezing, suggesting the multiple regulation of Snf1 on phospholipid metabolism. In the metabolism of glycerophospholipids, glycerol phospholipids, including PC, phosphatidylinositol (PI), phosphatidylethanolamine (PE), phosphatidylglycerol (PG), phosphatidic acid (PA), and their corresponding lysophospholipids (LysoPC, LysoPI, LysoPE, LysoPG, LysoPA), are related to signal transduction [46]. Some studies have shown that a decrease in the ratio of PC/PE can improve *S. cerevisiae* cell resistance to freezing stress [47, 48]. However, the mechanism of glycerophospholipid in the regulation of freezing response needs to be revealed.

## Conclusions

Summarily, overexpression of *SNF1* was effective to the improvement of the cell tolerance and leavening ability of baker’s yeast in freezing, which may be related to the dynamically upregulated proteasome pathway, altered metabolism of carbon sources and protectant molecules, and changed cell membrane components. Overexpression of *SNF1* could increase the content of 6 types of amino acids (including leucin, proline, serine, isoleucine, arginine, and homocitrulline), glycerol, and palmitic acid while decrease the content of LysoPC and LysoPE before freezing, probably conferring yeast cells strong resistance in freezing. After freezing, relative high level of 2 types of amino acids (including proline and lysine) and glycerol maintained by *SNF1* overexpression with increased content of LysoPC and LysoPE. To our knowledge, this study is the first attempt to investigate the impact of Snf1 in the freezing response of yeast and to explain the regulatory mechanism of Snf1 in freezing based on transcriptome and metabonomics profile. The findings will increase the knowledge of the regulatory effects of Snf1 protein kinase on the stress response of yeast. Meanwhile, the results enrich the knowledge of the cellular response of baker’s yeast cells to freezing stress and can direct future biochemical and molecular studies, which could improve our understanding of the stress response of yeast.

## Methods

### Strains and plasmids

The genetic characteristics of strains and plasmids are shown in Table 3.

**Table 3:** Characteristics of strains and plasmids used in the current study

Strains or plasmids	Relevant characteristic	Reference or source
Strains		
*Escherichia coli* DH5α	Φ80 *lacZΔM15 ΔlacU169 recA1 endA1 hsdR17 supE44 thi-1 gyrA relA1*	Yeast Collection Center of the Food Science and Technology Laboratory of Hainan University
*S. cerevisiae* ABY3	Industrial baker’s yeast	Yeast Collection Center of the Food Science and Technology Laboratory of Hainan University
ABY+YP	Yep-PK	This study
ABY+S	Yep-PSK	This study
Plasmids		
pUG6	*E. coli/S. cerevisiae shuttle vector*, *containing Amp*^+^, *loxP-KanMX-loxP* disruption cassette	Yeast Collection Center of the Food Science and Technology Laboratory of Hainan University
Yep-P	*URA3*^+^, *Amp*^*R*^* ori* control vector*, PGK1*_*P*_*-PGK1*_*T*_	[18]
Yep-PK	*KanMX*, *PGK1*_*P*_*-PGK1*_*T*_	
Yep-PSK	*KanMX*, *PGK1*_*P*_*-SNF1-PGK1*_*T*_	

### Media

*E. coli* was cultured in Luria–Bertani medium (consisted of 10 g/L tryptone, 10 g/L NaCl, and 5 g/L yeast extract) at 37 °C. To select positive *E. coli* transformants, 100 μg/mL ampicillin was used. The plasmid was obtained by the FastPure Plasmid Mini Kit (DC201, Vazyme, Nanjing, China).

*S. cerevisiae* was cultured in yeast extract peptone dextrose (YEPD) medium (consisted of 20 g/L peptone, 20 g/L glucose, and 10 g/L yeast extract) at 30 °C. To select positive *S. cerevisiae* transformants, 800 μg/mL G418 was used.

*S*. *cerevisiae* was statically pre-cultured in YEPD medium for 36 h at 30 °C. Then, the first pre-cultured cells were transferred to 200 mL YEPD medium at a 10% inoculation amount, rotated and shaken at 30 °C for 24 h at 180 rpm. The second pre-cultured cells were centrifuged at 1500*g* at 4 °C for 10 min and washed twice with 4 °C sterile water. To study the effect of *SNF1* overexpression on freeze resistance, 2 g fresh yeast cells (70% wet medium) were inoculated into low-sugar model liquid dough (LSMLD) medium, which was improved from the medium described by Panadero et al. [49]. The LSMLD medium was composed of 5 g/L of glucose, 2.5 g/L of (NH_4_)_2_SO_4_, 5 g/L of urea, 16 g/L of KH_2_PO_4_, 5 g/L of Na_2_HPO_4_, 0.6 g/L of MgSO_4_, 0.0225 g/L of nicotinic acid, 0.005 g/L of capantothenate, 0.0025 g/L of thiamine, 0.00125 g/L of pyridoxine, 0.001 g/L of riboflavin, and 0.0005 g/L of folic acid.

### Transformation of yeast

The plasmids Yep-PSK and Yep-PK were transferred to the parental strain ABY3 using lithium acetate/PEG procedure according to the previous study [38]. The transformants ABY+S and ABY+YP were verified by PCR using the primers PGK-F/PGK-R and K-F/K-R shown in Table 4.

**Table 4:** Primers used in the present study

Primer	Sequence (5′ → 3′)
PGK-F	TCTAACTGATCTATCCAAAACTGA
PGK-R	TAACGAACGCAGAATTTTC
K-F	CAGCTGAAGCTTCGTACGC
K-R	GCATAGGCCACTAGTGGATCTG
RT ACT1-F	ACGCTCCTCGTGCTGTCTTC
RT ACT1-R	GTTCTTCTGGGGCAACTCTCA
RT SNF1-F	CTGTCCCAGTCACCTCCAAC
RT SNF1-R	CTTGCCATCCTTCTTGCGTG

### Quantitative real-time polymerase chain reaction (qRT-PCR) analysis of *SNF1* gene expression

The cells were sampled from YEPD medium at 16 h, and the expression level of *SNF1* was tested according to the previous study [38]. The primers for the target gene *SNF1* and the reference gene *ACT1* are shown in Table 4.

### Determination of growth

*S*. *cerevisiae* cells were pre-cultured in YEPD medium at 30 °C to OD_600_ = 1.20, and transferred to 200 mL YEPD medium with a 4% inoculum. The cell density was measured at 30 °C using a UV spectrophotometer (T6, Persee, Beijing, China).

### Determination of freeze–thaw stress tolerance

After pre-culturation in 100 mL LSMLD medium for 30 min at 30 °C, 2 mL of fresh *S*. *cerevisiae* cultures were frozen at − 20 °C for 7 days. After thawing for 30 min in a 30 °C water bath, the frozen suspension was diluted properly and cultured on a YEPD plate for 2 days. The percentage of colony number after stress relative to that before stress was used to determine the cell survival rate. Three independent experiments were carried out.

### Determination of leavening ability

50 g of mixed lean dough (consisted of 140 g standard flour, 72.5 mL of water, 4.5 g of fresh yeast, and 2 g of salt) was weighed into a 250 mL graduated cylinder and pre-fermented at 30 °C for 30 min, then stored at − 20 °C for 7 days. After thawing at 30 °C for 30 min, CO_2_ amounts were recorded at 30 °C for 120 min. Three independent experiments were carried out.

### Transcriptome analysis

The cells (4 mg) were cultured in LSMLD medium for 30 min and sampled before and after freeze-thawing for transcriptome and metabonomics analyses.

Total RNA was extracted using the TRIzol method and mRNA was enriched using Oligo (dT) beads. The preparation of sequencing libraries and sequencing were conducted by Gene Denovo Biotechnology Co., Ltd (Guangzhou, China). One experiment was carried out.

After filtering the off-machine data to get clean and high-quality data, the reads were compared to the reference genome, and the transcripts were assembled by Cufflinks to obtain the known transcripts and new transcripts. Gene abundances were quantified by RSEM software [50]. The gene expression level was normalized using the Fragments Per Kilobase of transcript per Million fragments mapped (FPKM). The DEGs were identified using the edgeR package, and a fold change ≥ 2 and a false discovery rate (FDR) < 0.05 was considered to be a significant difference. The functional enrichment of the DEGs was analysed.

### Metabonomic analysis

The metabolites were extracted according to Yuan et al. [51]. Untargeted UPLC − QTOF-MS/MS was conducted according to Shu et al. [52] by LipidALL Technologies Co., Ltd (Changzhou, China). Four biological replicates were performed independently.

Data processing was performed according to Shu et al. [52]. The unknown differential metabolites were qualitatively identified by the comparison with standards of HMDB, METLIN, and other database, and *p* < 0.05 in the one-way analysis of variance (ANOVA) and a fold change > 1.5 were considered to be the significant difference.

## Statistical analysis

ANOVA and Least-Significant Difference (LSD) tests were used to determine the difference between the transformants and the parental strain in Figs 1, 2, 3, 4, and *p* < 0.05 was considered as significantly different.

## Supplementary Information

**Additional file 1**. Proteasome pathway information related to freezing stress. Differential expression between the *SNF1* overexpression transformant and the parental strain in proteasome (a) before freezing stress and (b) after freezing stress. The red and green arrows indicate upregulation and downregulation, respectively.

**Additional file 2**. Carbon metabolism pathway information related to freezing stress. Differential expression between the *SNF1* overexpression transformant and the parental strain in carbon metabolism (a) before freezing stress and (b) after freezing stress. The red and green arrows indicate upregulation and downregulation, respectively.

**Additional file 3**. Glycerol metabolism pathway information related to freezing stress. Differential expression between the *SNF1* overexpression transformant and the parental strain in glycerol metabolism (a) before freezing stress and (b) after freezing stress. The red and green arrows indicate upregulation and downregulation, respectively.

**Additional file 4**. Amino acid biosynthesis pathway information related to freezing stress. Differential expression between the *SNF1* overexpression transformant and the parental strain in amino acid biosynthesis (a) before freezing stress and (b) after freezing stress. The red and green arrows indicate upregulation and downregulation, respectively.

## Data Availability

All data generated or analysed during this study are included in this published article. The transcriptomics data are presented at https://www.ncbi.nlm.nih.gov/Traces/study/?acc=PRJNA681749. The metabolomics data are presented at https://data.mendeley.com/datasets/6jw4fnrcsm/1.
