# Supplementary figures and images for: Effect of overexpression of SNF1 on the transcriptional and metabolic landscape of baker’s yeast under freezing stress
Source: Microb Cell Fact. 2021 Jan 7;20:10. doi: 10.1186/s12934-020-01503-0 (PMC7792352; doi:10.1186/s12934-020-01503-0)

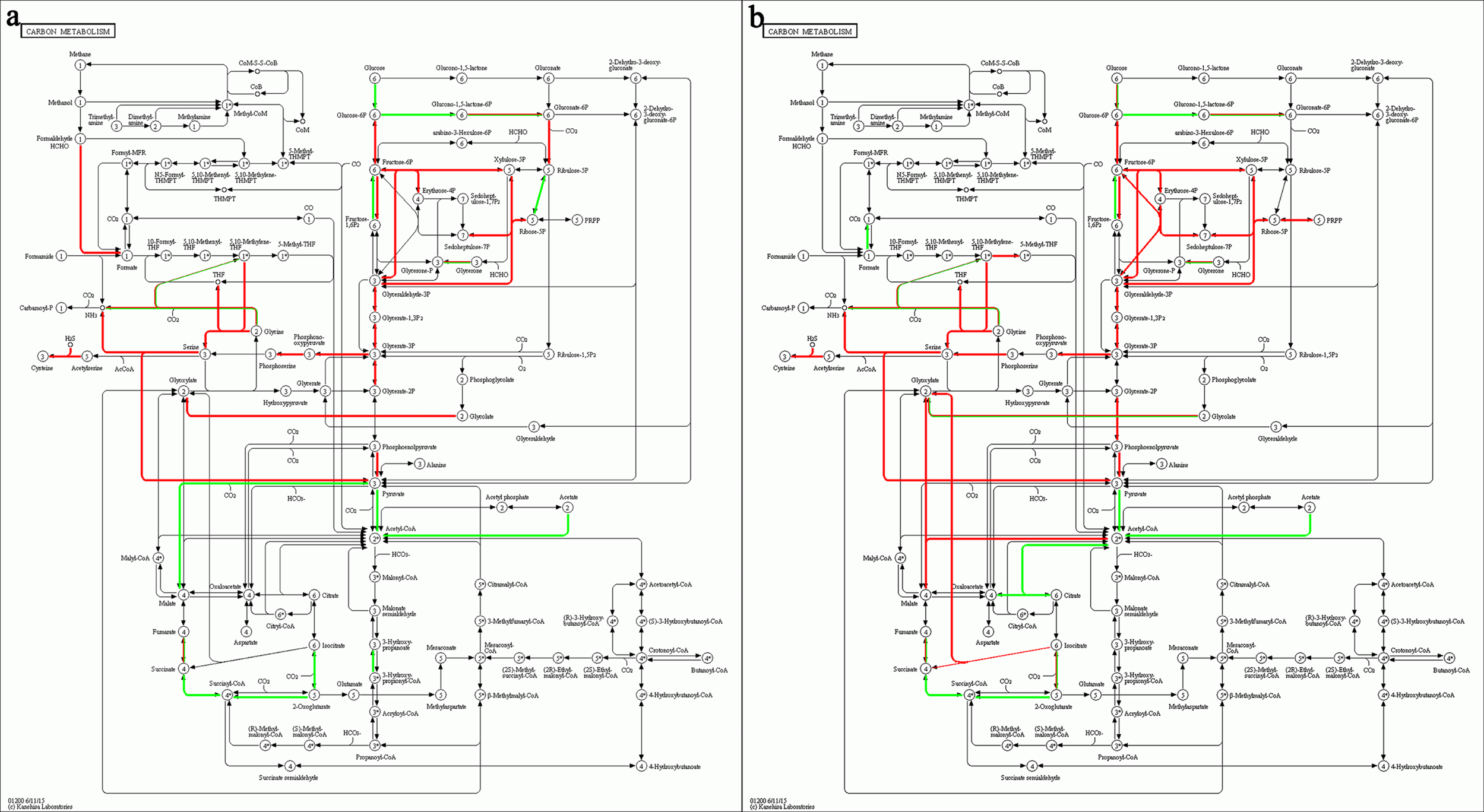

Supplement: Supplementary file 2 — Additional file 2. Carbon metabolism pathway information related to freezing stress. Differential expression between the SNF1 overexpression transformant and the parental strain in carbon metabolism (a) before freezing stress and (b) after freezing stress. The red and green arrows indicate upregulation and downregulation, respectively. [file 12934_2020_1503_MOESM2_ESM.tif]

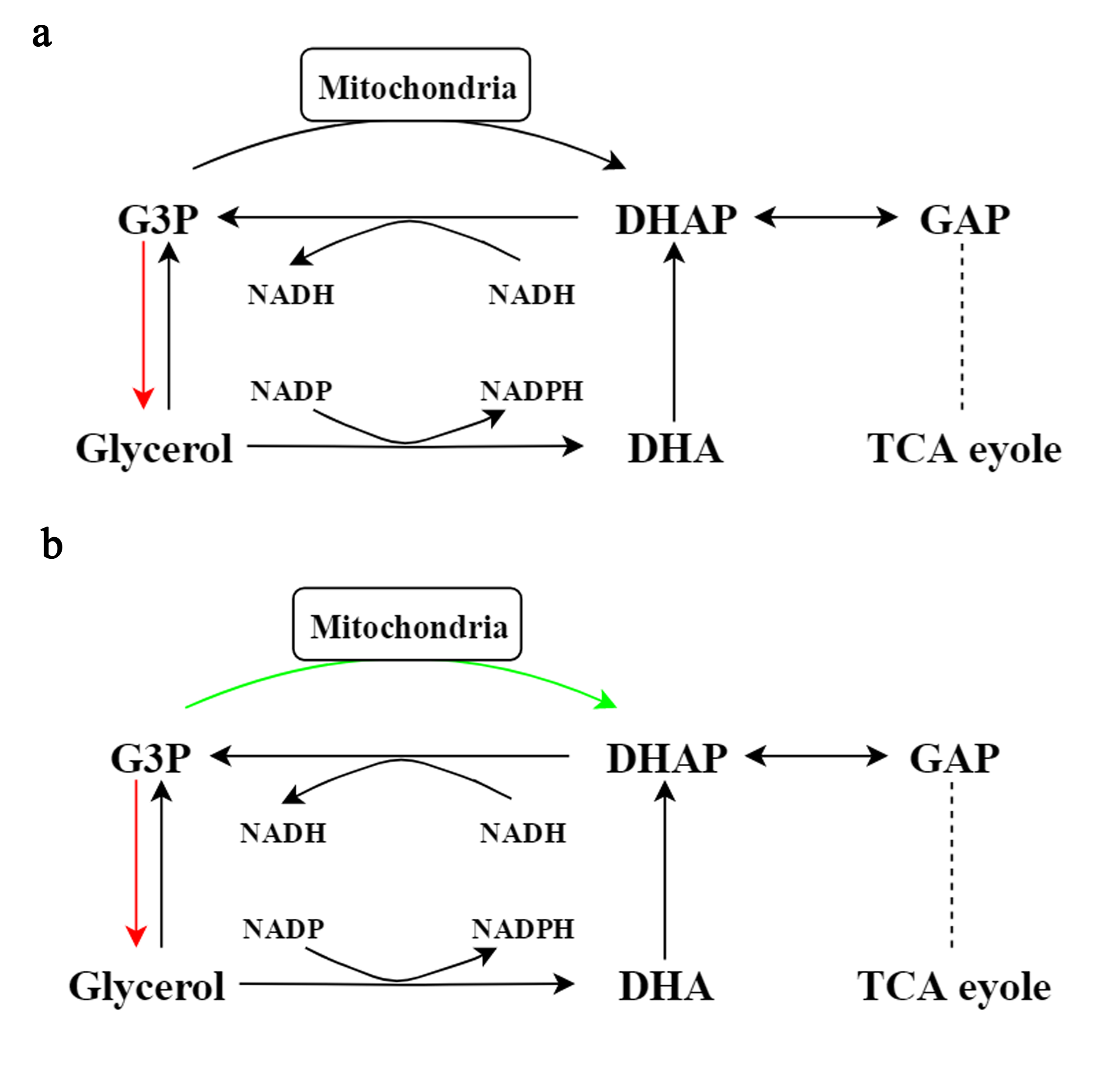

Supplement: Supplementary file 3 — Additional file 3. Glycerol metabolism pathway information related to freezing stress. Differential expression between the SNF1 overexpression transformant and the parental strain in glycerol metabolism (a) before freezing stress and (b) after freezing stress. The red and green arrows indicate upregulation and downregulation, respectively. [file 12934_2020_1503_MOESM3_ESM.tif]
